# Substrate Specificity and Allosteric Regulation of a d-Lactate Dehydrogenase from a Unicellular Cyanobacterium are Altered by an Amino Acid Substitution

**DOI:** 10.1038/s41598-017-15341-5

**Published:** 2017-11-08

**Authors:** Shoki Ito, Masahiro Takeya, Takashi Osanai

**Affiliations:** School of Agriculture, Meiji University, 1-1-1, Higashimita, Tama-ku, Kawasaki, Kanagawa 214-8571 Japan

## Abstract

Lactate/lactic acid is an important chemical compound for the manufacturing of bioplastics. The unicellular cyanobacterium *Synechocystis* sp. PCC 6803 can produce lactate from carbon dioxide and possesses d-lactate dehydrogenase (Ddh). Here, we performed a biochemical analysis of the Ddh from this cyanobacterium (*Sy*Ddh) using recombinant proteins. *Sy*Ddh was classified into a cyanobacterial clade similar to those from Gram-negative bacteria, although it was distinct from them. *Sy*Ddh can use both pyruvate and oxaloacetate as a substrate and is activated by fructose-1,6-bisphosphate and repressed by divalent cations. An amino acid substitution based on multiple sequence alignment data revealed that the glutamine at position 14 and serine at position 234 are important for the allosteric regulation by Mg^2+^ and substrate specificity of *Sy*Ddh, respectively. These results reveal the characteristic biochemical properties of Ddh in a unicellular cyanobacterium, which are different from those of other bacterial Ddhs.

## Introduction

Lactate/lactic acid is an organic acid used for the formation of poly(lactic acid) (PLA), which is a widely used biodegradable polyester^1,2^. Stereocomplex PLA is formed using enantiomeric PLA, poly(l-lactide) and poly(d-lactide), which enhances the mechanical properties, thermal stability, and hydrolysis resistance^3,4^. Lactate can be produced from petroleum; however, chemical synthesis generates a mixture of enantiomers. Optically pure l-lactate can be produced by large-scale microbial fermentation using, for example, *Lactobacillus* and *Bacillus* strains^5,6^. On the other hand, d-lactate production is required to produce stereocomplex PLA, but the process of d-lactate production has not been commercialised^7^. Thus, optically pure d-lactate production is important so that biorefinery can meet the demand for value-added bioplastic construction.

d-lactate is synthesised by NAD-dependent d-lactate dehydrogenase (Ddh, EC 1.1.1.28), whose biochemical properties have been studied using heterotrophic bacteria, including lactic acid bacteria^8–10^. Ddh catalyses oxidoreductase reactions between pyruvate and d-lactate using NADH as a co-factor. Ddh is phylogenetically distinguished from NAD-dependent l-lactate dehydrogenase (Ldh, EC 1.1.1.27) and belongs to a new group in the 2-hydroxyacid dehydrogenase family^11^. Generally, Ldh is allosterically activated in the presence of fructose-1,6-bisphosphate (FBP) by increasing substrate affinities to the enzymes^12,13^. Ddhs from Gram-negative bacteria, including *Fusobacterium nucleatum* and *Pseudomonas aeruginosa*, are activated by divalent cations such as Mg^2+^ ^14^. The *S*_0.5_ values of Ddhs from *Fusobacterium nucleatum* and *Pseudomonas aeruginosa* for pyruvate are reduced in the presence of Mg^2+^ ^14^. Ddhs from *Fusobacterium nucleatum*, *Pseudomonas aeruginosa*, and *Escherichia coli* are activated by FBP and citrate^14^. On the other hand, Ddh from *Escherichia coli* is not activated by Mg^2+^, demonstrating that the properties of Ddhs are diverse among Gram-negative bacteria^14^ Ddhs from Gram-positive bacteria, including *Pediococcus acidilactici* DSM 20284 and *Pediococcus pentosaseus* ATCC 25745, are hardly activated by divalent cations^15,16^. Thus, the allosteric regulation of lactate dehydrogenases is dependent on the species of bacteria.

Lactate production by heterotrophic bacteria requires external carbon sources such as glucose, which account for a large proportion of the production cost. Cyanobacteria, which perform oxygenic photosynthesis and fix CO_2_ via the Calvin-Benson cycle, have the potential to produce valuable products using CO_2_ as a carbon source. *Synechocystis* sp. PCC 6803 (hereafter *Synechocystis* 6803) is a unicellular, non-nitrogen fixing cyanobacterium that is widely used for basic research and contains Ddh (slr1556)^17^. *Synechocystis* cells can consume d-lactate under continuous light conditions^18^ and excrete d-lactate under dark, anaerobic conditions^19^. Biochemical analysis revealed that the Ddh from *Synechocystis* 6803 (*Sy*Ddh) is able to utilise both NADH and NADPH as cofactors^18^. *Sy*Ddh can catalyse pyruvate, hydroxypyruvate, glyoxylate, and d-lactate, but physiologically, it functions as a pyruvate reductase^18^. Ddh from *Lactobacillus delbrueckii* 11842 is a NADH-dependent dehydrogenase, and the substitution of three amino acid residues at positions 176~178 increases the *k*_cat_/*K*_m_ for NADPH by 184-fold^10^. For cyanobacteria, the key residues involved in allosteric regulation are unclear.

In this study, we performed a biochemical analysis of *Sy*Ddh and identified two amino acid residues that alter the specificities and affinities to substrates and allosteric regulation by Mg^2+^ of *Sy*Ddh.

## Results

### Affinity purification and biochemical characterisation of *Sy*Ddh

**Figure 1:**
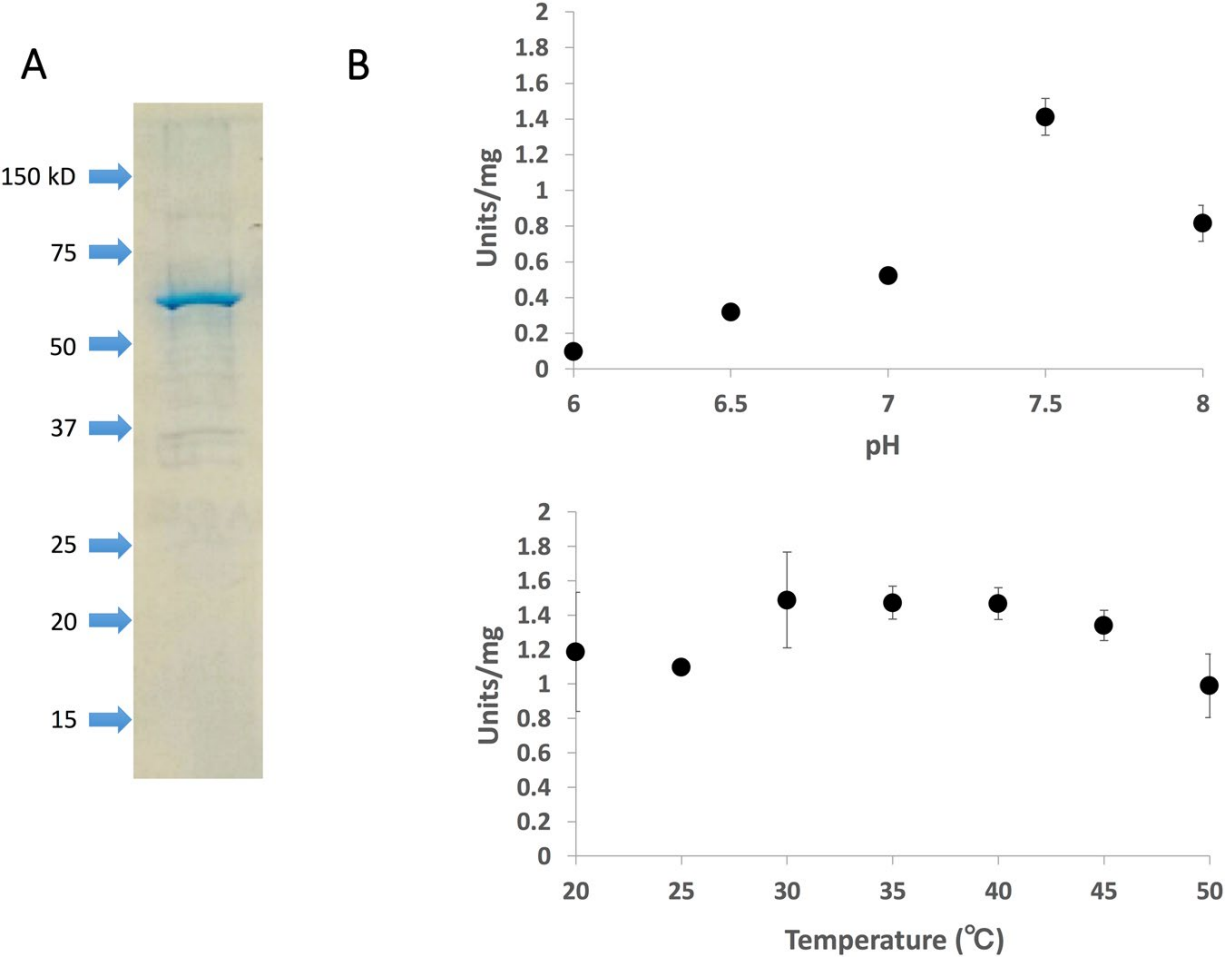
Biochemical analysis of *Synechocystis* 6803 d-lactate dehydrogenase (*Sy*Ddh). (**A**) Purification of GST-tagged *Sy*Ddh after separation by electrophoresis on a 12% SDS-PAGE gel. The gel was stained with InstantBlue reagent. Arrowheads indicate the molecular weight. (**B**) Effect of pH (top) and temperature (bottom) on *Sy*Ddh activity. Data represent the relative values of the means from three independent experiments. For the enzyme assay, 60 pmol (or 0.0038 mg) of *Sy*Ddh was used. One unit of *Sy*Ddh activity was defined as the consumption of 1 μmol NADH per minute.

To analyse the biochemical properties of *Sy*Ddh, glutathione-*S*-transferase-tagged *Sy*Ddh (GST-*Sy*Ddh) was expressed and purified from the soluble fraction of *Escherichia coli* cell extract by affinity chromatography (Fig. 1A). The enzymatic activity of *Sy*Ddh was highest at pH 7.5 (Fig. 1B). The enzymatic activities were similar at 30~40 °C (Fig. 1B). A subsequent enzymatic assay was performed at 30°C and pH 7.5. The *k*_cat_ value of *Sy*Ddh for pyruvate was 2.71 ± 0.26 s^−1^, and the *S*_0.5_ value of *Sy*Ddh for pyruvate was 0.38 ± 0.04 mM (Table 1).

**Table 1:** Kinetic parameters of *Sy*Ddhs for pyruvate and oxaloacetate at 30 °C and pH 7.5.

	*k*_cat_ (s^−1^)	*S*_0.5_ (mM)	*k*_cat_/*S*_0.5_ (s^−1^mM^−1^)	*K*_i_ (mM)	*n*_H_
**Pyruvate**					
*Sy*Ddh	2.71 ± 0.26	0.38 ± 0.04	7.14 ± 1.15		2.51 ± 0.11
*Sy*Ddh_Q14E	4.11 ± 0.71	0.52 ± 0.10	7.98 ± 0.35	16.51 ± 5.98	
*Sy*Ddh_S234G	1.71 ± 0.26	2.10 ± 0.18	0.82 ± 0.13		1.64 ± 0.10
**Oxaloacetate**					
*Sy*Ddh	2.16 ± 0.41	1.58 ± 0.76	1.55 ± 0.38	8.56 ± 3.39	
*Sy*Ddh_Q14E	2.38 ± 0.10	1.71 ± 0.38	1.46 ± 0.31		0.95 ± 0.12
*Sy*Ddh_S234G	1.27 ± 0.24	0.59 ± 0.05	2.19 ± 0.52	5.74 ± 1.04	

Parameters were calculated as described in the Materials and Methods. *k*_cat_, *S*_0.5_, and *k*_cat_/*S*_0.5_ are the turnover rate, half-maximum concentration giving rise to 50% *V*_max_, and catalytic efficiency, respectively. The inhibition constant (*K*_i_) and Hill coefficient (*n*_H_) are shown when the data exhibited substrate inhibition and cooperativity, respectively. Data represent the mean  ±  SD from three independent experiments.

Since Ddh has broad substrate specificity to 2-ketoacid in other bacteria, we also measured *Sy*Ddh activity using oxaloacetate as a substrate. *Sy*Ddh was able to catalyse not only pyruvate but also oxaloacetate as a substrate (Fig. S1); the *k*_cat_ value for oxaloacetate was 2.16 ± 0.41 s^−1^, and the *S*_0.5_ value of *Sy*Ddh for oxaloacetate was 1.58 ± 0.76 mM (Table 1). The *k*_cat_/*S*_0.5_ values of *Sy*Ddh for pyruvate and oxaloacetate were 7.14 ± 1.15 and 1.55 ± 0.38 s^−1^ mM^−1^, respectively (Table 1). Ddhs from lactic acid bacteria can catalyse phenylpyruvate^15,16,20,21^, but *Sy*Ddh could not catalyse phenylpyruvate as a substrate. The *k*_cat_ value for NADH was 2.60 ± 0.25 s^−1^, and the *S*_0.5_ value of *Sy*Ddh for NADH was 0.028 ± 0.002 mM, and therefore, *k*_cat_/*S*_0.5_ was 94.33 ± 7.83 s^−1^ mM^−1^. *Sy*Ddh had no activity with NADPH as a cofactor in our enzymatic assay.

We have tried to excise GST-tag from *Sy*Ddh using Factor Xa but the GST-tag was not excised. Then, *ddh* ORF was cloned into pGEX6P-1 and GST-*Sy*Ddh proteins were expressed and purified. GST-tag from pGEX6P-1 was excised by HRV 3C protease (Fig. S2A). However, purification after excision of the GST-tag was not succeeded (Fig. S2A). We measured enzymatic activities using the mixtures including *Sy*Ddh without GST-tag, but the relative activity did not increase. Therefore, we performed subsequent experiments using the proteins with GST-tags.

### Comparison of the amino acid sequence and the 3D structure of *Sy*Ddh

**Figure 2:**
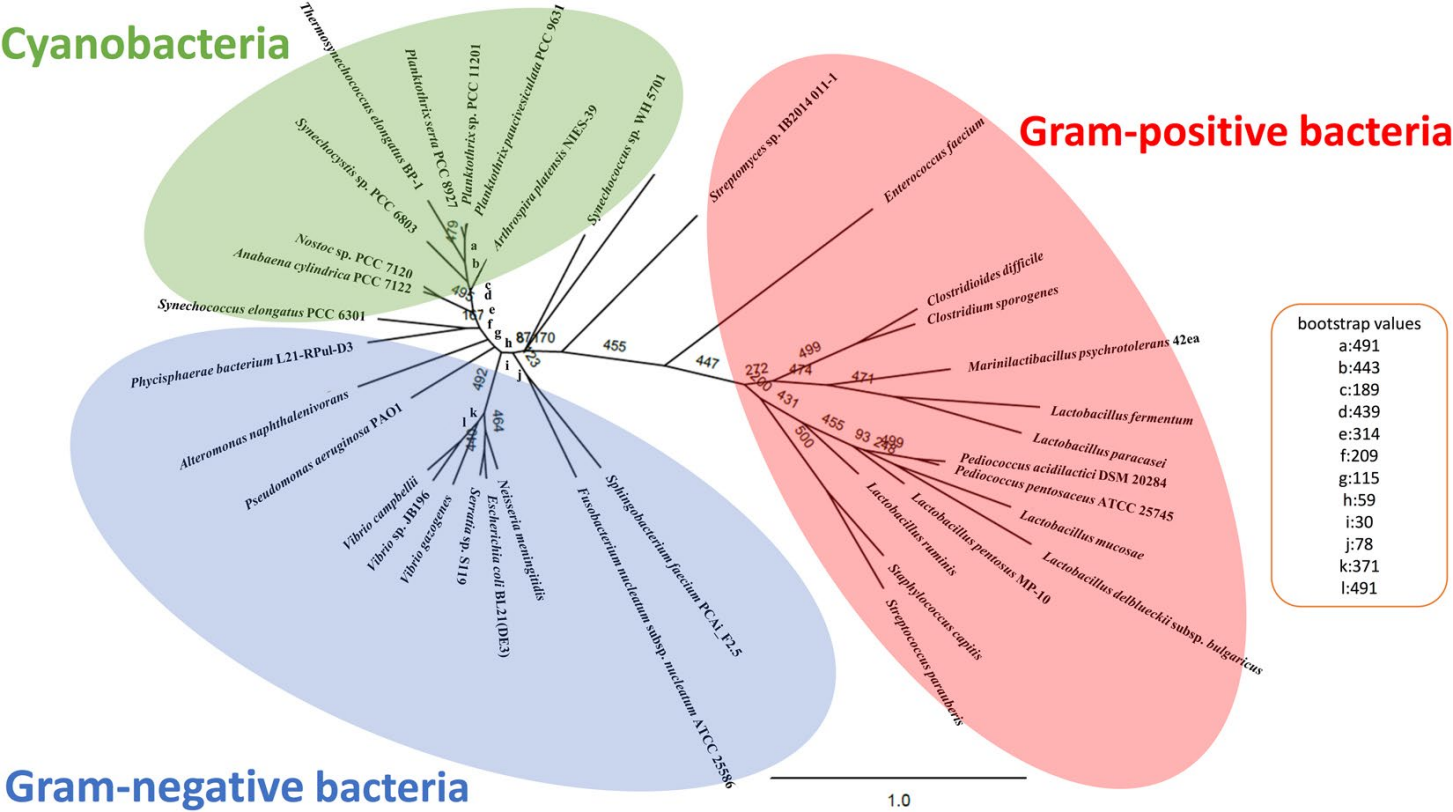
Phylogenetic analysis of the Ddhs from cyanobacteria, Gram-negative bacteria, and Gram-positive bacteria. Protein sequences and accession numbers were obtained from GenBank, followed by alignment using CLC Sequence Viewer software. A maximum-likelihood tree based on 262 conserved amino acids was generated using PHYML (http://www.atgc-montpellier.fr/phyml/). The bootstrap values were obtained from 500 replications.

We then compared the amino acid sequence of *Sy*Ddh with those of d-lactate dehydrogenases from Gram-negative and Gram-positive bacteria by generating a phylogenetic tree using the maximum-likelihood method (Fig. 2). The phylogenetic tree of 36 Ddhs revealed distinct clusters for Gram-negative and Gram-positive bacteria (Fig. 2). Cyanobacterial Ddhs were grouped with those from Gram-negative bacteria but were included in an independent clade (Fig. 2).

**Figure 3:**
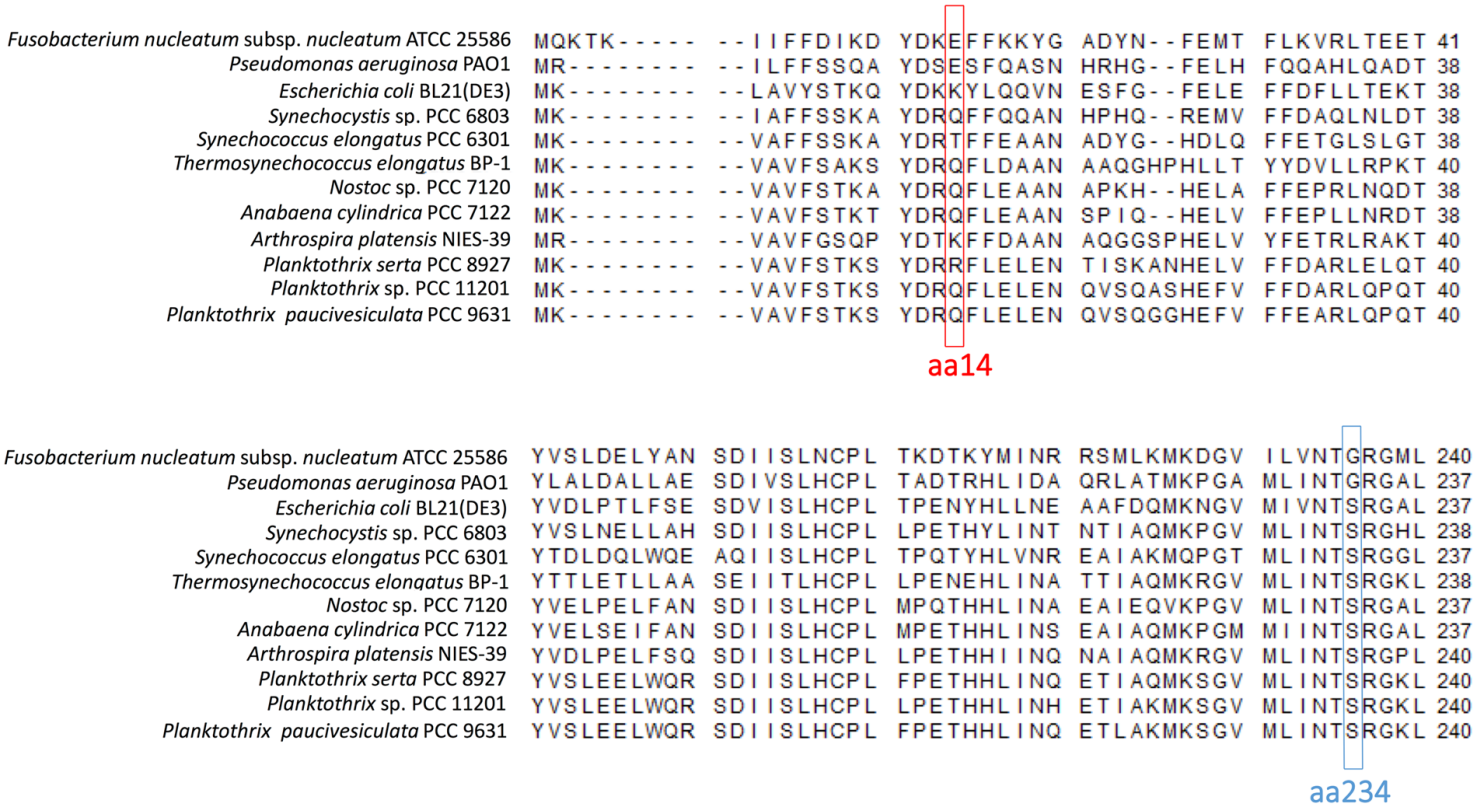
Multiple alignment of Ddhs from cyanobacteria and Gram-negative bacteria. The multiple protein sequence alignment was performed using CLC Sequence Viewer. The amino acid residues at positions 14 and 234 of *Sy*Ddh and equivalent residues of the other Ddhs are marked in red and blue, respectively.

**Figure 4:**
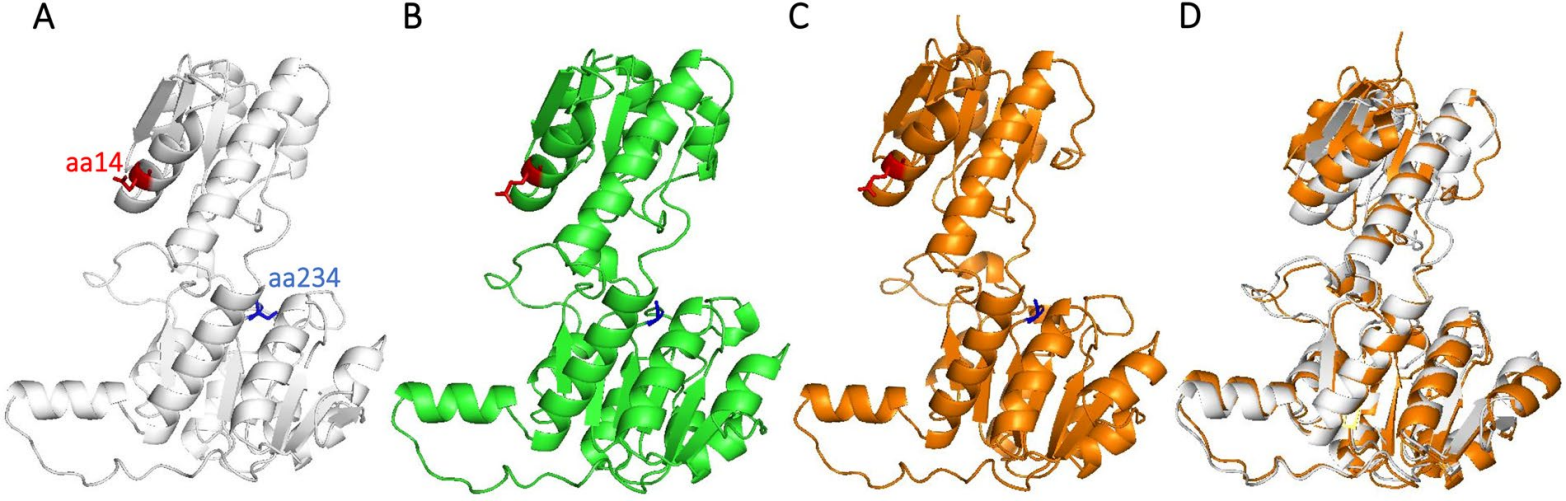
3D-structures of Ddhs represented by cartoon diagrams. (**A**) Structure of *Sy*Ddh generated by SWISS-MODEL. (**B**) 3D-structure of Ddh from *Pseudomonas aeruginosa* PAO1 (PDB ID: 3WWZ). (**C**) 3D-structure of Ddh from *Fusobacterium nucleatum* subsp. *nucleatum* ATCC 25586 (PDB ID: 3WWY). Amino acid residues at positions 14 and 234 of *Sy*Ddh and equivalent amino acid residues in Ddhs from *Pseudomonas aeruginosa* and *Fusobacterium nucleatum* were marked red and blue respectively. (**D**) Superposition of *Sy*Ddh and Ddh from *Fusobacterium nucleatum* subsp. *nucleatum* ATCC 25586.

Multiple sequence alignment analysis was then performed using nine Ddhs from cyanobacteria and three Ddhs from Gram-negative bacteria (Fig. 3). As mentioned above, Ddhs from *Fusobacterium nucleatum* and *Pseudomonas aeruginosa* have different biochemical properties from those of *E*. *coli* ^14^. Therefore, we searched for characteristic amino acid residues that were 1) conserved in *Fusobacterium nucleatum* and *Pseudomonas aeruginosa* but not in *E*. *coli* and 2) conserved in cyanobacteria. The amino acid residue glutamine at position 14 of *Sy*Ddh was relatively conserved among cyanobacteria, and the equivalent amino acid residues of *Fusobacterium nucleatum* and *Pseudomonas aeruginosa* Ddhs were glutamate (Fig. 3). The amino acid residue serine at position 234 of *Sy*Ddh was conserved among all cyanobacteria examined and in *E*. *coli*, while the equivalent amino acid residues of *Fusobacterium nucleatum* and *Pseudomonas aeruginosa* Ddhs were glycine (Fig. 3). To examine the location of amino acid residues at positions 14 and 234, we performed *in silico* analysis to predict 3D structure of *Sy*Ddh (Fig. 4A). *Sy*Ddh structure was generated from the 3D structures of *Pseudomonas aeruginosa* as a template (Fig. 4B). 3D structure of Ddh from *Fusobacterium nucleatum* was shown in Fig. 4C and superposition of *Sy*Ddh and Ddh from *Fusobacterium nucleatum* was described in Fig. 4D. These results indicate that *Sy*Ddh structure was similar to Ddhs from *Pseudomonas aeruginosa* and *Fusobacterium nucleatum* and amino acid residues at positions 14 and 234 of *Sy*Ddh and equivalent residues of Ddhs from *Pseudomonas aeruginosa* and *Fusobacterium nucleatum* were located at different domains (Fig. 4A~D).

### Amino acid substitutions altering the substrate specificity of *Sy*Ddh

**Figure 5:**
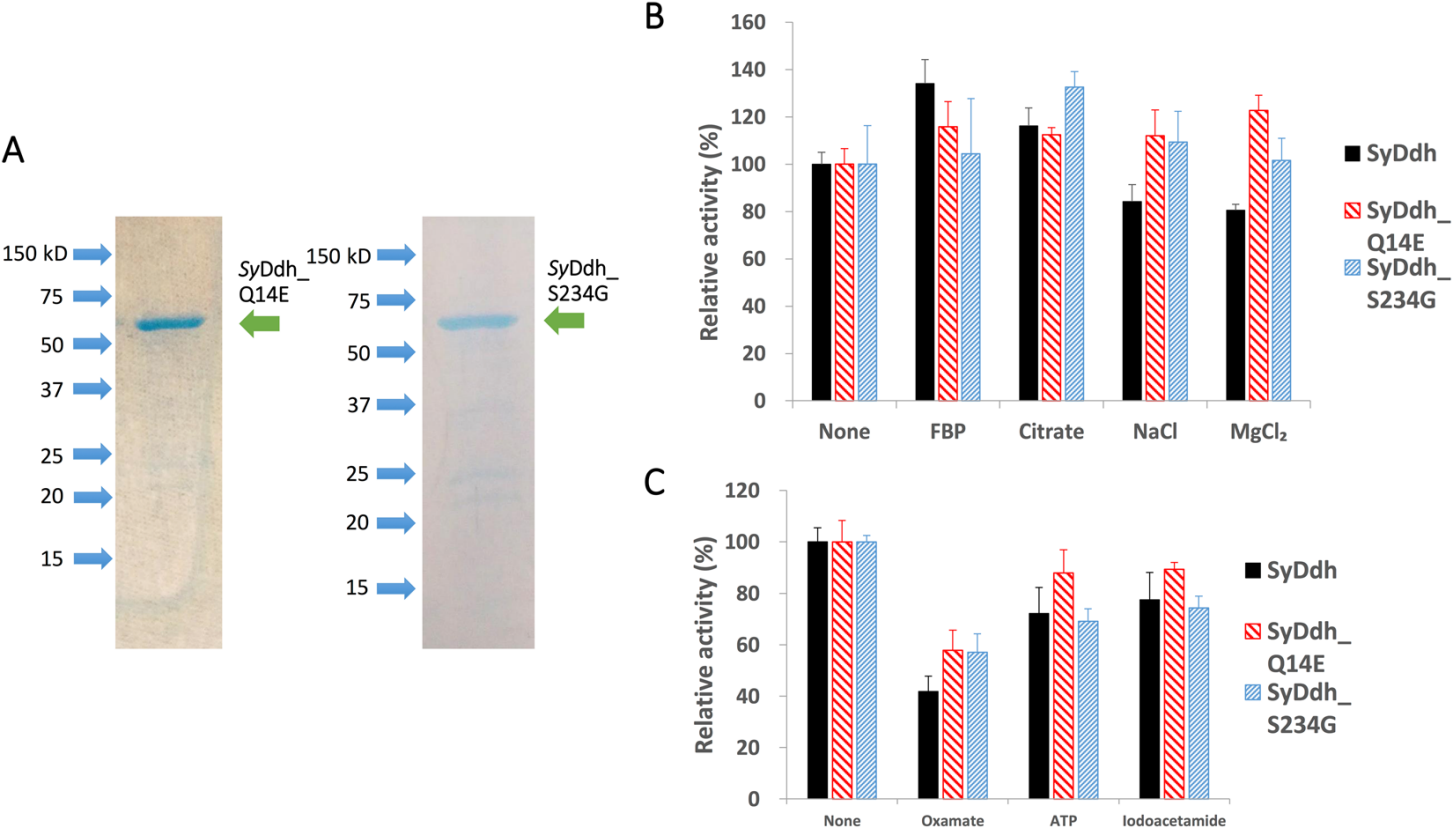
Enzymatic assay of *Sy*Ddh with a single substituted amino acid residue. *Sy*Ddh_Q14E and *Sy*Ddh_S234G are *Sy*Ddh with the glutamine at position 14 substituted with glutamate and with the serine at position 234 substituted with glycine, respectively. (**A**) Purification of GST-tagged *Sy*Ddh_Q14E and *Sy*Ddh_S234G after separation by electrophoresis on a 12% SDS-PAGE gel. The gel was stained with InstantBlue reagent. Arrowheads indicate the molecular weight. (**B** and **C**) Enzymatic activity in the presence of effectors. Each effector was added at a concentration of 2.5 mM. Ddh activity was measured at 30°C and pH 7.5 using 60 pmol *Sy*Ddhs. The graphs show the mean ± SD obtained from four independent experiments. Each activity of *Sy*Ddhs in the absence of effectors was set at 100%.

To clarify the role of the amino acid residues in *Sy*Ddh, we changed the glutamine residue at position 14 to glutamate (the protein was named *Sy*Ddh_Q14E) and the serine residue at position 234 to glycine (the protein was named *Sy*Ddh_S234G). The *Sy*Ddh_Q14E and *Sy*Ddh_S234G proteins were expressed similarly in *E*. *coli* as were *Sy*Ddh proteins, and the recombinant proteins were purified by affinity chromatography (Fig. 5A).

The *k*_cat_ values of *Sy*Ddh_Q14E for pyruvate and oxaloacetate increased to 4.11 ± 0.71 and 2.38 ± 0.10 s^−1^, respectively (Table 1). The *S*_0.5_ values of *Sy*Ddh_Q14E for pyruvate and oxaloacetate increased to 0.52 ± 0.10 and 1.71 ± 0.38 mM, respectively (Table 1). The *k*_cat_/*S*_0.5_ values of *Sy*Ddh_Q14E for pyruvate and oxaloacetate were 7.98 ± 0.35 and 1.46 ± 0.31 s^−1^ mM^−1^, respectively, which were similar to those of *Sy*Ddh (Table 1).

The *k*_cat_ values of *Sy*Ddh_S234G for pyruvate and oxaloacetate decreased to 1.71 ± 0.26 and 1.27 ± 0.24 s^−1^, respectively (Table 1). The *S*_0.5_ value of *Sy*Ddh_S234G for pyruvate increased to 2.10 ± 0.18 mM, while that for oxaloacetate decreased to 0.59 ± 0.05 mM (Table 1). The *k*_cat_/*S*_0.5_ value of *Sy*Ddh_S234G for pyruvate decreased to 0.82 ± 0.13 s^−1^ mM^−1^and that for oxaloacetate increased to 2.19 ± 0.52 s^−1^ mM^−1^ (Table 1).

*Sy*Ddh showed positive cooperativity (*n*_H_ = 2.51 ± 0.11) for pyruvate, and the Hill coefficient of *Sy*Ddh_S234G decreased to *n*_H_ = 1.64 ± 0.10 (Table 1). *Sy*Ddh_Q14E exhibited substrate inhibition by pyruvate, and the value of *K*_i_ was 16.51 ± 5.98 mM (Table 1). Contrary to pyruvate, *Sy*Ddh and *Sy*Ddh_S234G exhibited substrate inhibition by oxaloacetate, *K*_i_ = 8.56 ± 3.39 and 5.74 ± 1.04 mM, respectively (Table 1). *Sy*Ddh_Q14E did not show substrate inhibition by oxaloacetate, whose Hill coefficient was 0.95 ± 0.12 (Table 1).

### Amino acid substitution altered the biochemical properties in the presence of several effectors

**Figure 6:**
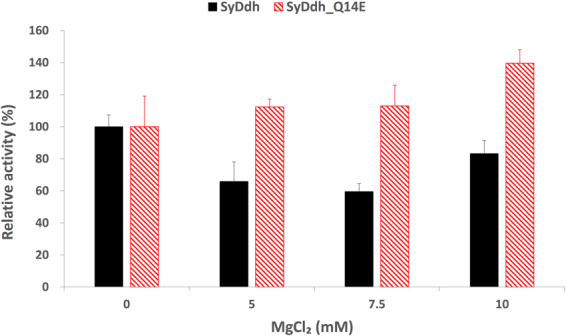
Enzymatic assay of *Sy*Ddh and *Sy*Ddh_Q14E with different Mg^2+^ concentrations. The graphs show the mean ± SD obtained from three independent experiments. For the enzyme assay, 60 pmol of *Sy*Ddhs was used. Each activity of *Sy*Ddhs in the absence of effectors was set at 100%.

The enzymatic activities in the presence of various effectors were then examined at 30°C and pH 7.5. *Sy*Ddh activity increased to 134% in the presence of 2.5 mM FBP, while those of *Sy*Ddh_Q14E and *Sy*Ddh_S234G were both lower and not activated by FBP (Fig. 5B). Citrate slightly increased *Sy*Ddh activity to 116% of the level with effectors, and *Sy*Ddh_Q14E and *Sy*Ddh_S234G were also both activated by citrate (Fig. 5B) The addition of 2.5 mM NaCl and MgCl_2_ reduced *Sy*Ddh activity to *ca* 80% of the level without effectors (Fig. 5B). *Sy*Ddh_S234G activity did not decrease in the presence of NaCl and MgCl_2_, and *Sy*Ddh_Q14E activity markedly increased particularly in the presence of MgCl_2_ (Fig. 5B). *Sy*Ddh activity decreased to 42% in the presence of 2.5 mM oxamate, and the activities of *Sy*Ddh_Q14E and *Sy*Ddh_S234G were similarly inhibited (Fig. 5C). *Sy*Ddh activity decreased to 72% and 78% in the presence of 2.5 mM ATP and 2.5 mM iodoacetamide respectively, and the activities of *Sy*Ddh_Q14E and *Sy*Ddh_S234G were similarly decreased in the presence of ATP and iodoacetamide (Fig. 5C). Increased concentrations of MgCl_2_ enhanced the activation of *Sy*Ddh_Q14E; the activity was enhanced up to 140% of the activity of *Sy*Ddh_Q14E in the absence of MgCl_2_ (Fig. 6).

The *k*_cat_ value of *Sy*Ddh for pyruvate in the presence of Mg^2+^ was 1.3 times that in the absence of Mg^2+^ (Tables 1 and 2). The *S*_0.5_ value of *Sy*Ddh for pyruvate in the presence of Mg^2+^ increased from 0.38 to 0.59 mM in the presence of Mg^2+^ (Tables 1 and 2). In case of *Sy*Ddh_Q14E, the *k*_cat_ value for pyruvate slightly decreased from 4.11 to 3.74 s^−1^; however, The *S*_0.5_ value decreased to one-fourth that in the presence of Mg^2+^ (Tables 1 and 2). The *k*_cat_/*S*_0.5_ value of *Sy*Ddh for pyruvate was reduced by Mg^2+^; on the contrary, that of *Sy*Ddh_Q14E markedly increased from 7.98 to 25.94 s^−1^ mM^−1^ (Tables 1 and 2).

**Table 2:** Kinetic parameters of *Sy*Ddhs for pyruvate in the presence of 2.5 mM MgCl_2_.

	*k*_cat_ (s^−1^)	*S*_0.5_ (mM)	*k*_cat_/*S*_0.5_ (s^−1^ mM^−1^)	*K*_i_ (mM)
*Sy*Ddh	3.53 ± 0.11	0.59 ± 0.05	6.00 ± 0.59	7.99 ± 0.93
*Sy*Ddh_Q14E	3.74 ± 0.22	0.14 ± 0.003	25.94 ± 1.09	

Parameters were calculated as described in the Materials and Methods. *k*_cat_, *S*_0.5_, and *k*_cat_/*S*_0.5_ are the turnover rate, half-maximum concentration giving rise to 50% *V*_max_, and catalytic efficiency, respectively. The inhibition constant (*K*_i_) is shown when the data exhibited substrate inhibition. Data represent the mean ± SD from three independent experiments.

## Discussion

In this study, we performed a biochemical analysis of Ddh in the unicellular cyanobacterium *Synechocystis* 6803 and demonstrated the substrate specificity and allosteric regulation of *Sy*Ddh, which were altered by amino acid substitutions (Tables 1 and 2).

The *k*_cat_ value of *Sy*Ddh for pyruvate was lower than those of Ddhs from other bacteria (Table 1). The *k*_cat_ values of Ddhs from other Gram-positive bacteria such as the lactic acid bacteria *Pediococcus acidilactici*, *Lactobacillus pentosus*, and *Pediococcus pentosaceus* are approximately 300 s^−1^ ^15,16,20^, while the *k*_cat_ value of Ddh from *Bacillus coagulans* was reported to be 23.6 s^−1^ ^21^. The *k*_cat_ values of Ddhs from the Gram-negative bacteria *Fusobacterium nucleatum* and *Pseudomonas aeruginosa* are approximately 400 s^−1^, while that from *E*. *coli* is 80 s^−1^ ^14^. Compared to these bacteria, the *k*_cat_ value of *Sy*Ddh was low (2.7 s^−1^) (Table 1), demonstrating that *Synechocystis* 6803 possesses inefficient d-lactate dehydrogenase. This result is consistent with other studies on lactate production using *Synechocystis* 6803 that have been performed by introducing the external lactate dehydrogenase from the lactic acid bacteria *Leuconostoc mesenteroides* ^18^ or the mutated glycerol dehydrogenase from *Bacillus coagulans* ^22^. Other groups have also succeeded in producing lactate using cyanobacteria by mutating the lactate dehydrogenases so that they use NADPH, not NADH, as a cofactor^23,24^ or constructing another biosynthetic pathway than dihydroxyacetone phosphate^25^. These studies indicate that internal lactate dehydrogenases in cyanobacteria are inefficient enzymes in view of metabolic engineering. The *S*_0.5_ value of *Sy*Ddh was similar to that of *Fusobacterium nucleatum* and lower than that of *E*. *coli* (Table 1)^14^, indicating that pyruvate affinity to *Sy*Ddh was similar or rather higher than that to other Ddhs in Gram-negative bacteria. Therefore, we here concluded based on biochemical evidence that *Sy*Ddh exhibited a lower *k*_cat_ value compared to Ddhs from other bacteria, which was the reason for the inefficiency of *Sy*Ddh (Table 1).

Aside from pyruvate, *Sy*Ddh was able to use oxaloacetate as a substrate (Fig. S1 and Table 1). Ddhs from *Fusobacterium nucleatum* and *Pseudomonas aeruginosa* also showed affinity to oxaloacetate, and the affinities of these Ddhs to oxaloacetate are higher than that of Ddh from *E*. *coli* ^14^. We found that the serine residue at position 234 of *Sy*Ddh is important for substrate specificity (Table 1). The *S*_0.5_ of *Sy*Ddh_S234G for pyruvate was 2.10 mM, which was 5.5 times of that of *Sy*Ddh (Table 1). On the contrary, the *S*_0.5_ of *Sy*Ddh_S234G for oxaloacetate was 0.59 mM, which was approximately one-third of that of *Sy*Ddh (Table 1), indicating that the serine residue at position 234 of *Sy*Ddh increased the affinity to pyruvate and decreased the affinity to oxaloacetate. For lactic acid bacteria, the amino acid residues at positions 52 and 296 of Ddh are important for the affinity to pyruvate^20,26^. Combined with their data, we conclude that Ddhs contain substrate flexibility, which can be altered by amino acid substitutions. Our biochemical analysis suggested that *Sy*Ddh is able to catalyse the reaction from oxaloacetate to malate. Previously, a metabolic engineering study showed that the disruption of *ddh* in *Synechocystis* 6803 decreased the production of not only lactate but also succinate under dark, anaerobic conditions^19^. Metabolic flux analysis has shown that succinate is produced by the reductive tricarboxylic acid cycle in this cyanobacterium^27^, and thus, these results indicate that *Sy*Ddh potentially catalyses the reaction from oxaloacetate to malate under dark, anaerobic conditions.

The value of the Hill coefficient of *Sy*Ddh for pyruvate was 2.51 (Table 1), demonstrating that *S*yDdh exhibits positive homotropic cooperativity with pyruvate. The Hill coefficient of *Sy*Ddh is similar to Ddhs from *Fusobacterium nucleatum* and *Escherichia coli* ^14^, which is consistent with our phylogenetic analysis (Fig. 2). Ddhs from these Gram-negative bacteria form homo-tetramer (Table 3), and therefore, *Sy*Ddh was predicted to form homo-tetramer, although our Blue Native PAGE could not show the quaternary structure (Fig. S2B). The enzymatic activity in the presence of effectors also differed between *Sy*Ddh and Ddhs from other bacteria; *Sy*Ddh was activated by FBP but repressed by divalent cations (Fig. 5B). The amino acid substitution at positions 14 and 234 altered *Sy*Ddh so that it was activated by divalent cations (Fig. 5B). Similar to Ddhs from *Fusobacterium nucleatum* and *Pseudomonas aeruginosa* ^14^, *Sy*Ddh_Q14E was markedly activated by Mg^2+^ due to the increased affinity to pyruvate (Table 2). Mg^2+^ may bind more strongly to *Sy*Ddh_Q14E than to *Sy*Ddh because glutamate contains a negative charge. Ddhs from Gram-positive bacteria are also diverse; the activity of Ddh in the genus *Pediococcus* is altered by metal ions, while that in the genus *Lactobacillus* is not^15,16,26,28^. Thus, biochemical properties of Ddhs are diverse, and we have demonstrated the substrate specificity and allosteric regulation of *Sy*Ddh and identified amino acid residues that are important for the biochemical properties in this cyanobacterium.

**Table 3:** A list of Ddhs from nine organisms showing the homology to *Sy*Ddh and predicted quaternary structure.

Species	PDB ID	Identity (%)	Oligo-State
*Pseudomonas aeruginosa* PAO1	3WWZ	59.75	Homo-tetramer
*Salmonella enterica* subsp. *enterica serovar* Typhi	4CUJ	53.37	Homo-tetramer
*Escherichia coli* BL21(DE3)	3WX0	51.52	Homo-tetramer
*Chlamydomonas reinhardtii*	4ZGS	49.23	Homo-tetramer
*Fusobacterium nucleatum* subsp. *nucleatum* ATCC 25586	3WWY	48.62	Homo-tetramer
*Aquifex aeolicus*	3KB6	36.62	Homo-tetramer
*Lactobacillus helveticus*	2DLD	34.08	Homo-dimer
*Lactobacillus delbrueckii* subsp. *bulgaricus*	1J49	33.75	Homo-dimer
*Sporolactobacillus inulinus* CASD	4XKJ	31.21	Homo-dimer

An amino acid sequence of *Sy*Ddh was used as a query and searched by SWISS-MODEL (https://www.swissmodel.expasy.org/).

## Methods

### Construction of the cloning vector and expression of recombinant proteins

The region of the *Synechocystis* 6803 genome containing the *ddh* (slr1556) ORF with the *Bam*HI-*Xho*I fragment was commercially synthesised and cloned into the *Bam*HI-*Xho*I site of pGEX5X-1 and pGEX6P-1 (GE Healthcare Japan, Tokyo, Japan) by Eurofins Genomics (Tokyo, Japan). Mutagenesis for amino acid substitution was performed by TakaraBio (Shiga, Japan). For *Sy*Ddh_Q14E and *Sy*Ddh_S234G, the regions +40–42 and +700–702 from the start codon in the *ddh* ORF were changed from CAA to GAA and AGT to GGT, respectively.

These vectors were transformed into *E*. *coli* DH5α (TakaraBio), and two litres of transformed *E*. *coli* were cultivated in LB media at 30°C with shaking (150 rpm), and expression of the protein was induced overnight in the presence of 0.01 mM isopropyl β-D-1-thiogalactopyranoside (Wako Chemicals, Osaka, Japan).

### Affinity purification of recombinant proteins

Affinity chromatography for protein purification was performed as described previously^29^. Two litres of DH5α cells was disrupted by sonication (model VC-750, EYELA, Tokyo, Japan) for 3~4 min at 20% intensity, and the disrupted cells were centrifuged at 5,800 × *g* for 2 min at 4°C. All the supernatant was transferred to 50-mL tubes on ice, and 560 μL of Glutathione-Sepharose 4B resin (GE Healthcare Japan, Tokyo, Japan) was mixed into the supernatant, followed by gentle shaking for 30 min. Then, 1 mM ATP and 1 mM MgSO_4_·7H_2_O were added to the mixture, which was incubated with gentle shaking for 40 min to remove intracellular chaperons. After centrifugation (5,800 × *g* for 2 min at 4°C), the supernatant was removed, and the resins were re-suspended in 700 μL of PBS-T (1.37 M NaCl, 27 mM KCl, 81 mM Na_2_HPO_4_·12H_2_O, 14.7 mM KH_2_PO_4_, 0.05% Tween-20) with 1 mM ATP/1 mM MgSO_4_·7H_2_O. After washing with PBS-T 10 times, the recombinant proteins were eluted with 700 μL of GST elution buffer (50 mM Tris-HCl, pH 8.0, 10 mM reduced glutathione) four times. The proteins were concentrated with a VivaSpin 500 MWCO 50000 device (Sartorius, Göttingen, Germany), and the protein concentration was measured with a PIERCE BCA Protein Assay Kit (Thermo Scientific, Rockford, IL). SDS-PAGE was performed to confirm protein purification with staining using InstantBlue (Expedion Protein Solutions, San Diego, CA). Proteases FactorXa and HRV 3C were purchased from Merck Millipore (Darmstadt, Germany) and TakaraBio, respectively. Blue Native PAGE was performed by applying the samples, each 9.4 μg of purified *Sy*Ddh proteins with 0.5% Brilliant Blue G (TCI, Tokyo, Japan), to NativePAGE™ 4–16% Bis-Tris Protein Gels (Thermo Scientific). Cathode buffer (50 mM Tricine-NaOH, 15 mM Bis-Tris/HCl, pH 7.0, 0.02% Brilliant Blue G) and anode buffer (50 mM Bis-Tris/HCl, pH 7.0) was used for electrophoresis. The gel was stained using InstantBlue after electrophoresis.

### Enzyme assay

Ddh activity was measured using 60 pmol of *Sy*Ddhs mixed in a 1 mL assay solution [100 mM potassium phosphate, 0.1 mM nicotinamide adenine dinucleotide hydride (NADH), 1 mM sodium pyruvate]. The absorbance at *A*_340_ was monitored using a Hitachi U-3310 spectrophotometer (Hitachi High-Tech., Tokyo, Japan). The kinetic parameters of Ddhs were calculated by curve fitting using Kaleida Graph ver. 4.5 software. When the data exhibited substrate inhibition, we used equation 1 ^30^. When the data exhibited cooperativity with a substrate, we used the Hill equation (equation 2)^31^. When the data showed neither substrate inhibition nor cooperativity, we used the Michaelis-Menten equation (equation 3).

1 $$v={V}_{max}[{\rm{S}}]/([{\rm{S}}]+{S}_{0.5}+{[{\rm{S}}]}^{2}/{K}_{{\rm{i}}})$$

2 $$v={V}_{max}{[{\rm{S}}]}^{n{\rm{H}}}/({[{\rm{S}}]}^{n{\rm{H}}}+{{S}_{0.5}}^{n{\rm{H}}})$$

3 $$v={V}_{max}[{\rm{S}}]/([{\rm{S}}]+{S}_{0.5})$$

*v* and *V*_max_ indicate reaction velocity and maximum reaction velocity, respectively. [S] indicates substrate concentration, and *S*_0.5_ indicates the half-maximum concentration giving rise to 50% *V*_max_. *K*_i_ is an inhibition constant, and *n*_H_ is the Hill coefficient. One unit of *Sy*Ddh activity was defined as the consumption of 1 μmol NADH per minute.

### *In silico* modelling

Homology modelling of *Sy*Ddh was performed with a database SWISS-MODEL (https://www.swissmodel.expasy.org/) using amino acid sequence of *Sy*Ddh from GenBank (Protein ID BAA18694.1) as a query. Ddh from *Pseudomonas aeruginosa* PAO1 (PDB ID: 3WWZ) was used as a template. 3D structures were visualized using a PyMOL software (v1.7.4, Schrödinger).

## Electronic supplementary material

Supplemental Figures
