# Supplementary material for: Substrate Specificity and Allosteric Regulation of a d-Lactate Dehydrogenase from a Unicellular Cyanobacterium are Altered by an Amino Acid Substitution
Source: Sci Rep. 2017 Nov 8;7:15052. doi: 10.1038/s41598-017-15341-5 (PMC5678113; doi:10.1038/s41598-017-15341-5)
Supplement: Supplementary file 1 — Supplemental Figures [file 41598_2017_15341_MOESM1_ESM.pdf]

Substrate Specificity and Allosteric Regulation of a D-Lactate Dehydrogenase from a Unicellular Cyanobacterium are Altered by an Amino Acid Substitution

Shoki Ito, Masahiro Takeya, Takashi Osanai*

[1]School of Agriculture, Meiji University, 1-1-1, Higashimita, Tama-ku, Kawasaki, Kanagawa 214-8571, Japan

Address correspondence to: Dr. Takashi Osanai, Meiji University. 1-1-1 Higashimita, Tama-ku, Kawasaki, Kanagawa 214-8571, Japan

Tel: +81-44-934-7103. Fax: +81-44-934-7103. E-mail: tosanai@meiji.ac.jp

Running Title: Biochemistry of cyanobacterial Ddh

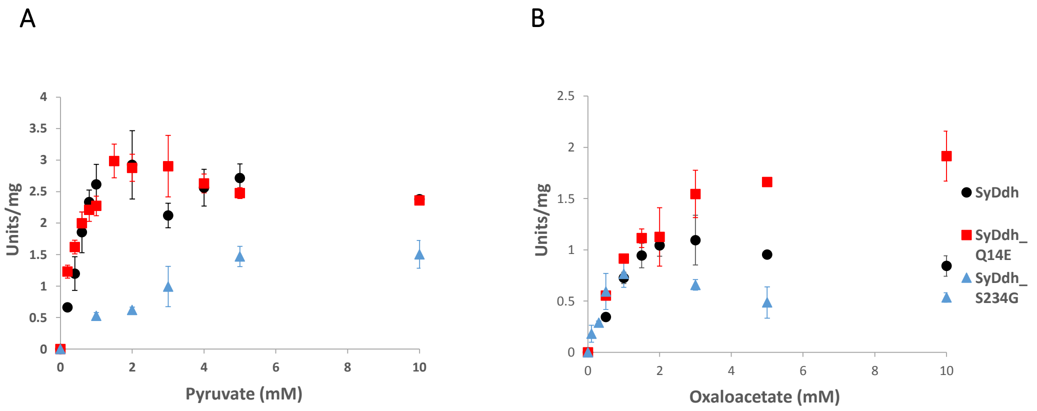

Figure S1. Saturation curves for *Sy*Ddh, *Sy*Ddh_Q14E, and *Sy*Ddh_S234G. Enzymatic activities were measured at 30°C, pH 7.5. Each 60 pmol of *Sy*Ddhs was used for enzymatic assay. (A) Pyruvate was used as a substrate for enzymatic assay. (B) Oxaloacetate was used as a substrate for enzymatic assay. The graphs show the mean ± SD obtained from three independent experiments.

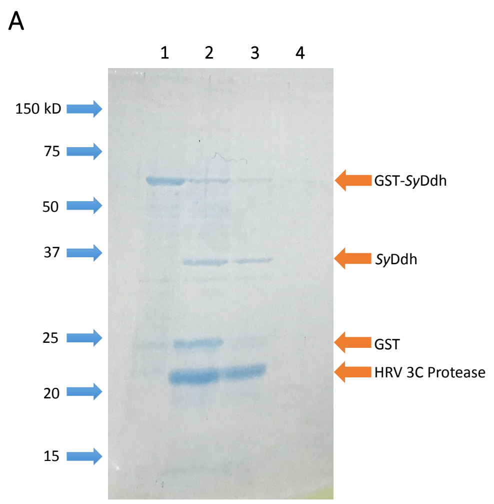

Figure S2. (A) SDS-PAGE of GST-*Sy*Ddh before and after HRV 3C Protease treatment. Lane 1: purified GST-*Sy*Ddh proteins. Lane 2: purified GST-*Sy*Ddh proteins excised by HRV 3C Protease. Lane 3: residual proteins after purification by Glutathione-Sepharose 4B resins. Lane 4: residual proteins after purification by TALON resins to remove HRV 3C Protease. Arrowheads indicate the molecular weight. (B) Blue Native PAGE of GST-*Sy*Ddh. 9.4 μg of purified GST-*Sy*Ddh proteins were separated by NativePAGE 4-16% Bis-Tris Protein Gels. Arrowheads indicate the molecular weight.
